# Comparison of maternal and fetal outcomes among patients undergoing cesarean section under general and spinal anesthesia: a randomized clinical trial: Comparação dos resultados maternos e fetais em pacientes submetidos a cesariana sob anestesia geral e raquianestesia: um ensaio clínico randomizado

**DOI:** 10.1590/1516-3180.2014.8901012

**Published:** 2014-12-19

**Authors:** Anıl İçel Saygı, Özkan Özdamar, İsmet Gün, Hakan Emirkadı, Ercüment Müngen, Yaşam Kemal Akpak

**Affiliations:** I MD. Attending Physician, Department of Obstetrics and Gynecology, Ankara Military Hospital, Ankara, Turkey.; II MD. Attending Physician, Department of Obstetrics and Gynecology, Gölcük Military Hospital, Gölcük, Kocaeli, Turkey.; III MD. Associate Professor, Department of Obstetrics and Gynecology, GATA Haydarpasa Training Hospital, Istanbul, Turkey.; IV MD. Attending Physician, Department of Anesthesiology and Reanimation, Gölcük Military Hospital, Gölcük, Kocaeli, Turkey.; V MD. Professor, Department of Obstetrics and Gynecology, GATA Haydarpasa Training Hospital, Istanbul, Turkey.

**Keywords:** Cesarean section, Anesthesia, general, Hemoglobins, Hematocrit, Anesthesia, spinal, Cesárea, Anestesia geral, Hemoglobinas, Hematócrito, Raquianestesia

## Abstract

**CONTEXT AND OBJECTIVE:**

As the rates of cesarean births have increased, the type of cesarean anesthesia has gained importance. Here, we aimed to compare the effects of general and spinal anesthesia on maternal and fetal outcomes in term singleton cases undergoing elective cesarean section.

**DESIGN AND SETTING:**

Prospective randomized controlled clinical trial in a tertiary-level public hospital.

**METHODS:**

Our study was conducted on 100 patients who underwent cesarean section due to elective indications. The patients were randomly divided into general anesthesia (n = 50) and spinal anesthesia (n = 50) groups. The maternal pre and postoperative hematological results, intra and postoperative hemodynamic parameters and perinatal results were compared between the groups.

**RESULTS:**

Mean bowel sounds (P = 0.036) and gas discharge time (P = 0.049) were significantly greater and 24^th^ hour hemoglobin difference values (P = 0.001) were higher in the general anesthesia group. The mean hematocrit and hemoglobin values at the 24^th^ hour (P = 0.004 and P < 0.001, respectively), urine volume at the first postoperative hour (P < 0.001) and median Apgar score at the first minute (P < 0.0005) were significantly higher, and the time that elapsed until the first requirement for analgesia was significantly longer (P = 0.042), in the spinal anesthesia group.

**CONCLUSION:**

In elective cases, spinal anesthesia is superior to general anesthesia in terms of postoperative comfort. In pregnancies with a risk of fetal distress, it would be appropriate to prefer spinal anesthesia by taking the first minute Apgar score into account.

**CLINICAL TRIAL REGISTRY:**

NTR17990

## RESUMO

**CONTEXTO E OBJETIVO:**

Como as taxas de partos cesáreos aumentaram, o tipo de anestesia na cesariana ganhou importância. Comparamos os efeitos da anestesia geral e da raquianestesia sobre os resultados maternos e fetais em casos de gestação única e no termo, com cesariana eletiva.

**TIPO DE ESTUDO E LOCAL:**

Ensaio clínico prospectivo, randomizado e controlado, em hospital público terciário.

**MÉTODOS:**

Estudo realizado com 100 pacientes que se submeteram a cesariana por indicação eletiva. As pacientes foram divididos aleatoriamente em grupos recebendo anestesia geral (n = 50) e raquianestesia (n = 50). Resultados maternos hematológicos pré e pós-operatórios, variáveis hemodinâmicas intra e pós-operatórias e resultados perinatais foram comparados entre os grupos.

**RESULTADOS:**

As médias de ruídos intestinais (P = 0,036) e tempo de descarga de gás (P = 0,049) foram significativamente mais elevadas e os valores de diferença de hemoglobina na 24^a^ hora (P = 0,001) foram maiores no grupo anestesia geral. Os valores médios de hematócrito e hemoglobina na 24^a^ hora (P = 0,004 e P < 0,001, respectivamente), o volume de urina na primeira hora de pós-operatório (P < 0,001) e a pontuação mediana de Apgar no primeiro minuto (P < 0,0005) foram significativamente maiores, e o tempo até o primeiro requerimento de analgésicos também foi significativamente maior (P = 0,042) no grupo raquianestesia.

**CONCLUSÃO:**

Nos casos eletivos, raquianestesia é superior à anestesia geral em termos de conforto pós-operatório. Em gestações com risco de sofrimento fetal, seria adequado preferir raquianestesia, levando em conta o Apgar no primeiro minuto.

## INTRODUCTION

The frequency of cesarean section births continues to steadily rise worldwide.^1^ Even though the cesarean procedure has become very safe over the years, it is still associated with high rates of maternal and perinatal mortality and morbidity.^2^ The overall postoperative morbidity rate associated with cesarean births is 35.7%.^3^ The higher mortality and morbidity rates might be attributable not only to the surgical procedure but also to the anesthetic technique preferred.

Cesarean anesthesia has gained importance as the cesarean birth rates have increased. For many years, general anesthesia was the preferred type for use in cesarean procedures.^4^ Although it has many advantages, such as faster induction, better cardiovascular stability with lower incidence of hypotension, and good control over ventilation, use of anesthetic drugs that cross the placental barrier can nevertheless produce neonatal depression.^4^ Moreover, complications such as maternal aspiration syndrome and intubation failure, which may occur during general anesthesia and contribute towards maternal mortality, have been reported.^5^^,^^6^

Thus, recently, the rates of cesarean section using regional anesthesia have been increasing and regional anesthesia has now become the preferred anesthetic technique for avoiding both maternal and fetal complications.^7^^,^^8^ Although many reports have shown that regional anesthesia and general anesthesia have almost identical indexes of neonatal wellbeing,^9^^,^^10^ ^11^^,^^12^ a growing number of anesthesiologists prefers regional anesthesia under elective conditions. Regional anesthesia-related hypotension due to sympathetic blockade may affect neonatal short-term outcomes by impairing uteroplacental perfusion.^13^ Additionally, cerebrospinal fluid (CSF) leakage following lumbar puncture may induce headache, nausea and vomiting.^7^^,^^14^^,^^15^ On rare occasions, insufficiency of the regional blockade and consequent conversion to general anesthesia has been reported.^1^ As a result, no optimal cesarean technique and no ideal anesthetic method for minimizing the surgical morbidity among candidate mothers has yet been described in the literature. Today, the choice of anesthesia depends on the mother’s request, obstetric reasons and the anesthesiologist’s experience level.

## OBJECTIVE

Our aim in this study was to compare maternal pre/postoperative hematological parameters, maternal intra/postoperative hemodynamic parameters and postpartum newborn results in term, singleton, non-complicated pregnancies that underwent elective cesarean section under spinal and general anesthesia.

## METHODS

This study was approved by the ethics committee/institutional review board (number B.30.2.İST.0.30.90.00/7724, Cerrrahpaşa School of Medicine) and it conforms to the provisions of the Declaration of Helsinki (as revised in Tokyo 2004). This prospective randomized, controlled clinical trial involved 100 singleton pregnant women, between 18 and 35 years of age, who delivered at term (37-40 weeks) by means of elective cesarean section, in a tertiary public university hospital between January 2011 and October 2011. All the patients were in the physical condition classified as 1 or 2 according to the American Society of Anesthesiologists (ASA) classification.

Patients presenting the following were not included in the study: requirement for emergency caesarean section for delivery; classification as ASA status ≥ III; multiple gestations; great multiparity (more than four deliveries); macrosomia (≥ 4500 grams); polyhydramnios (defined as amniotic fluid index more than 25 cm); placental abnormalities, such as placental abruption, placenta previa or adherent placenta; possibility of high risk of intraoperative hemorrhage, such as cases of placenta previa or coagulation defects; premature membrane rupture; preterm delivery (defined as before the 37^th^ week of pregnancy); post-term delivery (defined as pregnancies exceeding the 40^th^ gestational week); pregnancies with obstetric problems such as fetal anomaly; intrauterine growth restriction (defined as birth weight two standard deviations below the population mean for gestational age and sex); oligohydramnios (defined as amniotic fluid index less than 5 cm); pre-eclampsia; gestational diabetes mellitus; height less than 150 cm; body mass index (BMI) ≥ 30 kg/m^2^; allergy caused by local anesthesia; systemic illnesses such as goiter, diabetes mellitus or anemia (Hb < 8 g%); and unsuitability for regional anesthesia.

In all patients, the pregnancy was dated based on confirmation of the last menstrual period or was redated from the first or second-trimester ultrasound examination. All the patients’ basic demographic information was recorded and each patient was given a study information sheet and a consent form was signed. No pharmacological premedication was administered to the patients.

The cases included in the study were randomly enrolled into either the general anesthesia group (n = 50) or the spinal anesthesia group (n = 50) by means of drawing lots from a bag that had been prepared in the operating room before the operation began. Intravenous accesses were established for all the patients and prehydration with 1000 ml of colloid solution was started. Additionally, routine standard monitoring was performed (electrocardiogram monitoring, noninvasive follow-up of arterial blood pressure and follow-up of the peripheral oxygen saturation).

Before the implementation of general anesthesia, in the cases in the general anesthesia group, pre-oxygenation was performed using 100% oxygen for five minutes. Subsequently, 4-5 mg/kg of thiopental was used to induce anesthesia. After establishing muscle relaxation using 0.8 mg/kg of rocuronium intravenously (IV), endotracheal intubation was performed through cricoid pressure. For all cases, controlled ventilation (Datex-Ohmeda S/5 Avance, GE Healthcare, United States) was established by setting the tidal volume to 8-10 ml/kg and respiration frequency to 10-12 minutes. Anesthesia was maintained with a mixture of 1-1.5% sevoflurane and 50% nitrous oxide in oxygen. If a maintenance dose was required, muscle relaxation was established with 0.15 mg/kg of rocuronium. At the end of the surgery, the residual neuromuscular block was antagonized by means of neostigmine (30 µg/kg) and atropine (15 µg/kg). In order to minimize the risk of aspiration, the patients were extubated once they were awake.

Before the operation, the patients in the spinal anesthesia group were rapidly given 1000 ml of colloid solution at the rate of 15 ml/kg for 20 minutes. Afterwards, following skin cleansing done in the sitting position, the subarachnoid space was entered using a 25-gauge needle from the L3-4 or L4-5 interspace. After the cerebral spinal fluid (CSF) flow had been observed, 2.2 ml of 0.5% hyperbaric bupivacaine was administered into the subarachnoid space at a rate of 0.1 ml/s.

The spinal anesthesia patients were lateralized to the left for 5-10 minutes in a fully supine position. The patient’s head was then elevated to 30 degrees, thus placing her in an appropriate position. The motor block level was determined from the Bromage scale and the sensory block level was determined by means of a hot/cold test, as a dermatome level. When the sensory block reached a sufficient level (T4-T5), the operation was started. After the newborn had been delivered, the patients were sedated using midazolam when required.

In both groups, for those who presented hypotension (mean arterial blood pressure falling below 60 mmHg) following the anesthesia, 1.5 ml/kg of crystalloid solution, in addition to colloid solution, was firstly implemented. In case of continuation of hypotension, ephedrine hydrochloride (5-10 mg; IV) was implemented.

For patients who developed bradycardia (heart rate falling below 50 beats per minute), 0.5 mg of atropine sulfate IV was administered. Cases of oxygen saturation (SpO_2_) lower than 90%, as detected using pulse oximetry, were deemed to present desaturation and 100% O_2_ was administered at the rate of 4 l/m, through a face mask.

After applying the respective anesthesia to the groups, a standard lower-segment transverse uterine incision was made. The placenta was removed manually. After the newborn and the placenta had been taken out, 0.2 mg/ml of methylergobasine maleate intramuscularly and 1 g of prophylactic second-generation cephalosporin IV were administered to all patients. Twenty units of oxytocin were added to the IV solution and it was administered at a rate of 125 ml/h.

The assessment on the newborn was made by a pediatrician who was present in the operating room. Information about the newborn (existence of meconium, sex of the newborn, his/her weight, first and fifth minute Apgar scores, information about hospitalization in the pediatric clinic and indications for hospitalization) was all recorded.

All the cesarean deliveries were performed in a standard fashion under the control of surgeons who had at least 10 years of experience. The myometrial incision was closed as double-layer continuous suturing with a 1-0 polyglycolic acid suture (Vicryl; Ethicon) and the layers of visceral peritoneum were brought together. In the general anesthesia group, when the skin closure began, 0.5 mg/kg of tramadol HCl was administered IV. The duration of the operation, from beginning the skin incision to applying the last skin closure suture, was recorded.

The postoperative treatment was also similar for each group. For the first hour of the postoperative stage, the patients were monitored in the anesthesia intensive care unit. Systolic blood pressure (SBP) in the 30^th^ and 60^th^ minutes, diastolic blood pressure (DBP), mean arterial blood pressure (MAP), heart rate (HR), first-hour peripheral oxygen saturation (SpO_2_) and first-hour urine output were recorded. Before the patients were transported from the anesthesia intensive care unit to the clinic, all of them were assessed using the verbal rating scale (VRS), which indicated the severity of postoperative pain on a scale of scores between 0 = no pain and 10 = worst pain ever. When VRS was ≥ 4, 75 mg of diclofenac sodium (Voltaren) was implemented intramuscularly. Subsequently, the patients were transferred to the obstetrics clinic and whenever VRS was ≥ 4, analgesic was administered. The time at which the first dose of analgesic was required and the total number of doses administered within the first 24 postoperative hours were recorded.

Postoperatively, all the patients received 3 l of intravenous fluid containing oxytocin (10 IU/l) over the first 24 hours following delivery and were mobilized within the first 6 hours. All the patients were allowed oral liquid intake, particularly water, from the 6^th^ postoperative hour onwards, but were only allowed to have aqueous food intake within the first 24 hours in order to facilitate the return of gastrointestinal functions.

Within the first 24 hours, the times that elapsed until postoperative bowel action (subsequent to the anamnesis received from the patient and the auscultation examination) and gas discharge, and body temperature measurements (over the first 24 postoperative hours, at least two measurement values were > 38.5 °C), were recorded in relation to all the patients. In addition, hemoglobin and hematocrit values were determined both before and in the 24^th^ hour following the surgery.

Statistical analyses were performed using the Statistical Package for the Social Sciences 15.0 software for Windows (SPSS, Chicago, IL, USA). Descriptive statistics were stated as the mean, standard deviation, frequency and percentage. Statistical analyses were performed using Student’s t test for continuous variables and the chi-square test for categorical variables. Multivariate logistic regression was performed to assess the independence of the associations by adjusting for potential confounding factors. Previous abdominal operations, body mass index (BMI) at delivery, maternal age at delivery, week of pregnancy at the time of delivery and smoking status were used for multivariate logistic regression models as potential confounders that might be correlated with bowel sounds, gas discharge, need for analgesics, total dose of analgesics, postoperative fever, postoperative Apgar scores at first and fifth minutes and admission to the neonatal intensive care unit (NICU). For each potential confounder, we calculated adjusted odds ratios and the 95% confidence interval (CI). Statistical significance was defined as P < 0.05.

## RESULTS

A total of 100 patients were included in the study. The patients were divided into two groups of 50 each, named the general anesthesia and spinal anesthesia groups, according to the route of administration of anesthesia. The most frequent indication for cesarean delivery was a previous cesarean and, among all the patients, its rate was 53%. The maternal demographic characteristics and perinatal outcomes of the two groups are shown in Table 1.

**Table 1.**
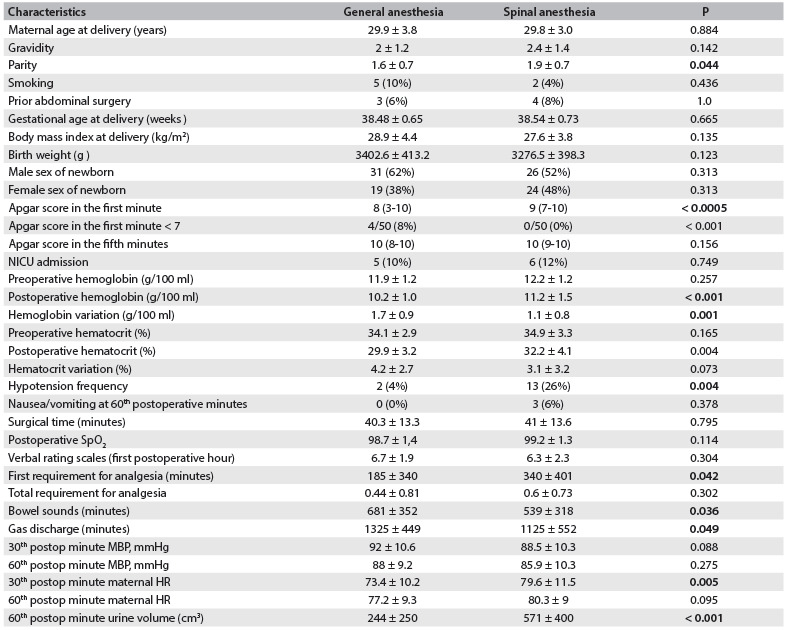
Comparison of demographic characteristics and perinatal, maternal hematological and postoperative monitoring outcomes

Data are presented as number or number (percent) and mean ± standard deviation except for the Apgar scores which are expressed as median (range). Postop = postoperative; MBP = mean blood pressure; HR = heart rate

As seen in Table 1, the results seemed to be similar except that the first minute Apgar scores were significantly better and the parity was significantly higher in Spinal Anesthesia Group. In the general anesthesia group, there was only one baby with a first minute Apgar score less than 5, whereas no baby had a fifth minute Apgar scores less than 7. Regarding the first minute APGAR scores of the newborns, after adjustments were made by taking confounders into account, the difference between two groups continued to exist (P = 0.005).

The NICU admission rates were similar in the two groups (10% and 12% respectively; P = 0.749). In the spinal anesthesia group, the risk was 1.227 times greater than that in the general anesthesia group. After making adjustments by taking confounders into account, the NICU admission risk in Spinal Anesthesia Group was 1.536 times greater than that of General Anesthesia Group. Nevertheless, in terms of NICU admission rates, there was no statistically significant difference between the two groups (P = 0.538). Only one newborn in the spinal anesthesia group was admitted to the NICU due to meconium aspiration, while respiratory problems constituted the other reasons for NICU admission. None of the neonates stayed in the NICU more than 5 days.

Comparisons of the patients’ hematological and hemodynamic parameters are presented in Table 1. It can be seen that the preoperative hemoglobin and hematocrit values of the two groups were similar (P = 0.257 and P = 0.165, respectively), while the values 24 hours after the operation were significantly lower in the general anesthesia group than in the spinal anesthesia group (P < 0.001 and P = 0.004, respectively). Regarding the change from before to after the operation, only the difference in hemoglobin values was greater in the general anesthesia group (1.7 ± 0.9 versus 1.1 ± 0.8; P = 0.001). A requirement for blood transfusion due to postpartum hemorrhage appeared only in the general anesthesia group, in one patient. Significant hypotension following induction of anesthesia was observed in the spinal anesthesia group (2% and 13%, P = 0.004; relative risk [RR]: 4.24; 95% CI: 1.15-15.60). In assessing the maternal hemodynamic parameters, the hazard ratio (HR) values at the 30^th^ minute were significantly higher in the spinal anesthesia group (73.4 ± 10.2 versus 79.6 ± 11.5; P = 0.005). The urine output in the 60^th^ minute after the operation was significantly lower in the general anesthesia group (244 ± 250 versus 571 ± 400; P < 0.001).

Although there was no statistically significant difference in VRS scores, it was observed that these scores were lower in the spinal anesthesia group (6.7 ± 1.9 versus 6.3 ± 2.3; P = 0.304). However, the total analgesic requirement was lower in the general anesthesia group (0.44 ± 0.81 versus 0.6 ± 0.73; P = 0.302). In parallel with the results from the pain scale, a requirement for analgesics was seen over a significantly longer span of time in the spinal anesthesia group than in the general anesthesia group (185 ± 340 versus 340 ± 401; P = 0.042).

The starting times for bowel sounds (681 ± 352 versus 539 ± 318; P = 0.036) and for gas discharge (1325 ± 449 versus 1125 ± 552; P = 0.049) were significantly longer in the general anesthesia group than in the spinal anesthesia group. After corrections were made by taking confounders into account, it was observed that there was still a significant difference between the two groups in terms of the starting time of bowel sounds, irrespective of the confounders (P = 0.05), while there was no difference between these groups in terms of the time that elapsed until gas discharge (P = 0.103). It was observed that smoking might have led to this statistically significant difference (P = 0.003).

## DISCUSSION

Even today, despite increasing knowledge and skills, cesarean delivery still carries higher maternal and perinatal mortality and morbidity risks than does vaginal delivery.^16^ These risks can be attributed not only to the emergency status of the operation and the jeopardy brought about by the surgical technique, but also to the potential hazards produced by the anesthetic method. Today, there is still no unquestionably recognized and ideal cesarean technique nor is there a single anesthetic method, although the global trend is shifting towards regional anesthesia.^7^ In a study conducted in the UK, the regional anesthesia rate was 69.4% in 1992, while it reached 94.9% in 2002.^8^

A number of factors have played a role in the rise of regional anesthesia rates, such as the increasing experience of anesthesiologists, the fact that newborns do not get exposed to the depressant effect relating to inhalation agents, the low rate of risk of lung aspiration, increasing sociocultural level, the fact that the mother is awake after the cesarean delivery and early establishment of the bond between mother and newborn, given that the mother can see her baby shortly after birth.^17^^,^^18^ Today, general anesthesia is preferred in emergency obstetric situations, such as cord prolapse, in which there is a need for swift and reliable induction, and also bleeding placenta previa and uterus inversion.^7^

Regional anesthesia is divided into two subgroups: epidural anesthesia and spinal anesthesia. A careful examination of the relevant literature reveals that there is no difference between epidural and spinal anesthesia in terms of maternal side effects.^19^ Epidural anesthesia is preferred because it has unlimited duration and postoperative pain management;^20^ spinal anesthesia, on the other hand, is preferred because of its advantages of being implemented in a shorter span of time, having faster onset of action and requiring less medication, and its capacity to form a strong sensory and motor block.^19^ The cases in which regional anesthesia is definitely contraindicated are serious maternal hypotension, skin infection and maternal coagulopathy. In the relevant literature, the rates of conversion to general anesthesia are given as approximately 1 in 100 cases.^1^

Over recent years, our clinic has also experienced an increase in the number of cases of regional anesthesia in comparison with general anesthesia in elective cesarean deliveries. One of the frequent maternal complications of spinal anesthesia is intraoperative hypotensive episodes,^19^^,^^21^^,^^22^ and the potential risk factors for this are increased sympathetic tonus, advanced age, obesity, high-level block,^23^ insufficiency of the volume of fluid given before induction,^24^ fixed drug dose administration for induction instead of adjusting it specifically to the person in question^25^ and increased cerebrospinal pressure.^26^ Ephedrine has been proposed for lessening the frequency of hypotension,^27^^,^^28^ but it has been suggested recently in the literature that although prophylactic use of ephedrine and phenylephrine were both effective in preventing maternal hypotension during cesarean under spinal anesthesia, phenylephrine was superior to ephedrine in treating hypotension with higher umbilical cord blood pH values.^29^^,^^30^^,^^31^ In our routine clinical practice, we use colloid solutions for pre-hydration and in cases of hypotension, our first-line interventions are to increase the colloid rate and provide supplementation with crystalloid solutions. Ephedrine is used if the hypotension is resistant to therapy. In the recent literature, it is stressed that crystalloid coload is more effective than preload for prevention of maternal hypotension after spinal anesthesia.^32^

The real question is whether maternal intraoperative hypotensive episodes increase neonatal mortality by impairing uteroplacental function.^13^^,^^22^^,^^33^ A general examination of the current literature indicates that it is the duration and severity of hypotension that accounts for neonatal mortality.^22^^,^^34^^,^^35^ Maayan-Metzger et al.^22^ stated that despite very high prevalence of maternal hypotension during cesarean sections, term infants tend to tolerate this placental blood perfusion challenge without any major sequel.

Regarding elective cesarean delivery cases described in the literature, there is no difference between the general and spinal anesthesia groups in terms of average first and fifth-minute Apgar scores.^9^ The Cochrane database analyses also confirm this finding, pointing out that there is no significant difference between these two groups, not only in terms of the first and fifth-minute average Apgar scores, but also in terms of the newborn’s oxygen requirement.^36^ Nevertheless, the proportion of newborns with Apgar scores ≤ 6 has been found to be significantly low in the first minute in the spinal group, but without any difference in scores between the two groups with regard to the fifth minute.^37^

In our study, the frequency of occurrence of intraoperative hypotension in the spinal anesthesia group was found to be 26%, which was lower than the rates given in the current literature.^9^^,^^18^ The lower rates may be attributable to sufficient fluid infusion before induction, appropriate patient position and ephedrine use as a vasopressor agent. The neonatal outcomes were similar between the groups except for the first minute Apgar scores of 8 (range: 3-10) and 9 (range: 7-10) (P < 0.0005).

To our knowledge, there are very few published papers focusing on postpartum maternal hemorrhage, return of gastrointestinal functions, infection and postpartum analgesic requirement, with a view to comparing cesarean deliveries performed by means of general and spinal anesthesia. Maternal hemorrhage has been reported to be less frequent with spinal anesthesia,^36^ and some researchers have found that the need for blood transfusion is higher in the general anesthesia group.^38^ All these findings have been correlated with two potential causes: first, general anesthetic agents suppress uterine contractions; and second, these agents impair platelet functions and hemostasis.^39^

In our study, both postoperative hemoglobin values (10.2 ± 1.0 versus 11.2 ± 1.5, P < 0.001) and hematocrit values (29.9 ± 3.2 versus 32.2 ± 4.1, P = 0.004) were significantly lower in the general anesthesia group. Blood transfusion was needed after postpartum atonic bleeding in only one patient. In the literature, there has only been one study comparing the effects of different anesthesia techniques for cesarean section on gastrointestinal function.^40^

One of the most important findings of the present study was the early recovery of gastrointestinal function following spinal anesthesia. Also in our study, bowel sounds (681 ± 352 versus 539 ± 318, P = 0.036) and gas discharge (1325 ± 449 versus 1125 ± 552, P = 0.049) occurred statistically significantly earlier in the spinal anesthesia group.

The same study as mentioned above is the only one in the literature comparing spinal and general anesthesia in terms of the time until the first requirement for analgesia.^40^ In the abovementioned study, the time until the first postoperative requirement for analgesia in the spinal anesthesia group was longer. Cochrane database analyses also indicate the analgesic requirements that appear later in cases of use of regional and particularly epidural anesthesia.^36^ In our study also, the time until the first postoperative requirement for analgesia in the spinal anesthesia group was longer (185 ± 340 versus 340 ± 401, P = 0.042). Nevertheless, there was no significant difference between these two groups in terms of total requirement for analgesia (0.44 ± 0.81 versus 0.6 ± 0.73, P = 0.302).

Spinal anesthesia is as effective as general anesthesia. Maternal hypotension can be managed successfully with modest doses of ephedrine and IV fluid infusions. The factors that influence the selection of anesthesia method in cesarean delivery are the following: whether the operation is an emergency; any systemic problems; the patient’s choice; and the experience level of the anesthesiologist.

## CONCLUSIONS

We are of the opinion that spinal anesthesia is superior to general anesthesia in terms of fetal wellbeing. Furthermore, with regard to pregnancies with fetal problems, we consider that it would be more appropriate to prefer the method of spinal anesthesia by taking first minute Apgar scores into account. Additionally, considering the later appearance of postoperative requirement for analgesia and the faster return of gastrointestinal function, spinal anesthesia emerges as the anesthetic method-of-choice for cesarean sections.
